# Where in the Cell Are You? Probing HIV-1 Host Interactions through Advanced Imaging Techniques

**DOI:** 10.3390/v8100288

**Published:** 2016-10-19

**Authors:** Brennan S. Dirk, Logan R. Van Nynatten, Jimmy D. Dikeakos

**Affiliations:** Department of Microbiology and Immunology, Schulich School of Medicine and Dentistry, The University of Western Ontario, London, ON N6A 5C1, Canada; bdirk@uwo.ca (B.S.D.); lvannyna@uwo.ca (L.R.V.N.)

**Keywords:** cell culture, microscopy, HIV-1, host-pathogen interactions

## Abstract

Viruses must continuously evolve to hijack the host cell machinery in order to successfully replicate and orchestrate key interactions that support their persistence. The type-1 human immunodeficiency virus (HIV-1) is a prime example of viral persistence within the host, having plagued the human population for decades. In recent years, advances in cellular imaging and molecular biology have aided the elucidation of key steps mediating the HIV-1 lifecycle and viral pathogenesis. Super-resolution imaging techniques such as stimulated emission depletion (STED) and photoactivation and localization microscopy (PALM) have been instrumental in studying viral assembly and release through both cell–cell transmission and cell–free viral transmission. Moreover, powerful methods such as Forster resonance energy transfer (FRET) and bimolecular fluorescence complementation (BiFC) have shed light on the protein-protein interactions HIV-1 engages within the host to hijack the cellular machinery. Specific advancements in live cell imaging in combination with the use of multicolor viral particles have become indispensable to unravelling the dynamic nature of these virus-host interactions. In the current review, we outline novel imaging methods that have been used to study the HIV-1 lifecycle and highlight advancements in the cell culture models developed to enhance our understanding of the HIV-1 lifecycle.

## 1. Introduction

From the beginnings of life on Earth, organisms have been both plagued and befriended by nanometer sized intruders, commonly known as viruses, which take refuge within the depths of cells. Although viruses often cause asymptomatic infections, certain viruses have the capacity to ravage populations in order to complete their infectious lifecycle. Yet, such obligate intracellular parasites—which depend on the host cells which they infect to replicate—are the simplest, smallest and most abundant biological agents in the tree of life [1]. Undoubtedly, the success of any virus lies in its ability to effectively complete its lifecycle. However, this covert invasion of viruses into host cell territory is combatted by the immune system.

Perhaps the epitome of all viral infections is the human immunodeficiency virus (HIV). Upon entry, the HIV-1 RNA genome is reverse-transcribed into DNA, following which it may integrate into host chromatin to establish chronic infection, promote replication of the viral genome, and direct transcription and translation of viral mRNA to produce the viral proteins required for viral assembly and exit [2]. Indeed, HIV-1 represents a virus that infects the very immune cells required to eliminate it from the human body and may lie latent within the genetic code of the host organism, manifesting as a silent and chronic infection [2]. As the host immune system attempts to attack the virus shielded within our cells, our immune cells are destroyed in the process, eventually resulting in acquired immunodeficiency syndrome (AIDS) [2]. Very few viruses are able to establish an infection as persistent as HIV-1, and as such, HIV-1 has evolved to effectively infiltrate the inner workings of the host cell from the point of viral entry to the point of viral exit during its lifecycle like no other virus before.

Undoubtedly, to fully elucidate the behaviour of HIV-1 and develop novel therapeutics, it is valuable to examine the movement of viruses and the interactions of their proteins within the cell. Fortunately, there is an equal army of microscopic techniques readily equipped to capture a glimpse of the infectious cycle when applied in conjunction with cell culture models. To visualize viral entry, cellular hijacking, as well as assembly and exit, a wide variety of cell-culture based imaging techniques exist. Indeed, it is such imaging methods in cell culture that have unraveled the profoundly intricate molecular dance of viral proteins hijacking host cells and transforming cells into factories for their own replication. Here we discuss the forms of microscopy available for these imaging purposes, as well as the specific manipulations of viruses and cells in culture that can be implemented to experimentally evaluate virus-host cell encounters. 

## 2. Breaking and Entering: Imaging Mechanisms of HIV-1 Entry

In order to initiate the viral lifecycle in a susceptible host, the viral particle must be able to enter the host cell; an event referred to as cell-free viral entry. In brief, free HIV-1 enters the cell by engaging its viral envelope glycoprotein 120 (GP120), with both the primary entry receptor, cluster of differentiation 4 (CD4), and the co-receptors, C-C chemokine receptor type 5 (CCR5) or C-X-C chemokine receptor type 4 (CXCR4), on the host cell [3,4]. Subsequent to this receptor engagement, the viral fusion peptide glycoprotein 41 (GP41) undergoes a conformational shift permitting the fusion of the viral envelope and the host cell membrane [5]. This fusion event creates a pore resulting in a route of entry for the HIV-1 nucleocapsid to enter the cytoplasm [6]. Indeed, fusion at the plasma membrane has long been perceived to be the primary mode of entry. However, recent reports [7,8] have demonstrated that a subset of viral particles are initially endocytosed and subsequently fuse with a post-plasma membrane compartment, the endosome, in order to gain cytoplasmic entry. With recent advances in cell culture models and live cell imaging, we are now equipped to understand the full complexity of HIV-1 entry.

**Figure 1:**
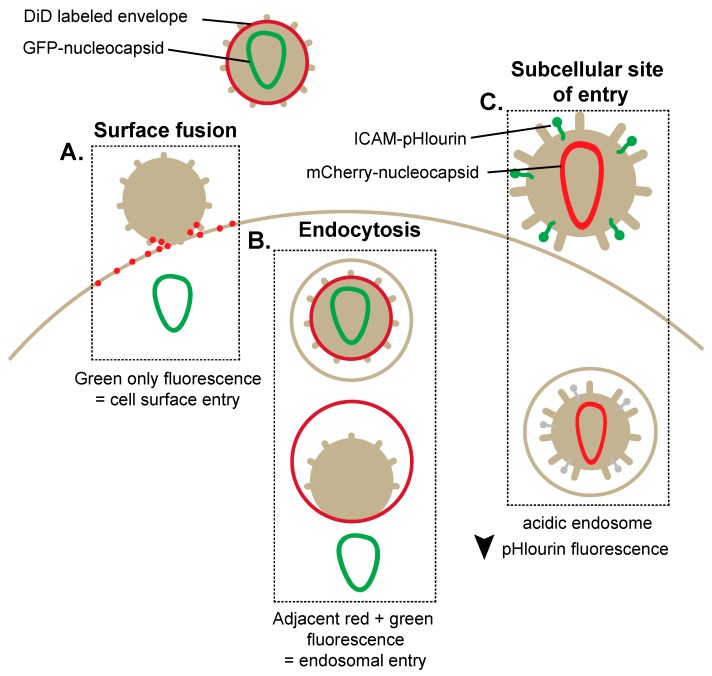
Fluorescent human immunodeficiency virus type 1 (HIV-1) vectors enable the visualization of alternative methods of HIV-1 cell-free entry. (**A**) Dialkylcarbocyanine analogue (DiD)-labeled HIV-1 expressing green fluorescent protein (GFP)-tagged nucleocapsid identifies viral fusion at the plasma membrane. Plasma membrane fusion corresponds to a loss of **red** fluorescence due to diffusion of the DiD probe in the plasma membrane and the subsequent presence of **green** fluorescence; (**B**) Endosomal entry of HIV-1 is marked by red and green puncta within the cell as DiD probe diffusion is limited to the smaller endosomal location. Viral entry through an endosome is detected by the visualization of a **green** signal (GFP-tagged nucleocapsid) migrating away from a **red** signal (DiD-labeled endosome); (**C**) pH-dependent HIV-1 entry can be measured by incorporation of an intracellular adhesion molecule 1 (ICAM)-pHlourin-labeled HIV-1. The decrease of pHlourin fluorescence corresponds to the presence of HIV-1 within the increasingly acidic endosomal compartment.

In particular, the ability to engineer viruses with fluorescent transgenes has become a valuable tool for studying the HIV-1 lifecycle. Notably, the advent of mono, di and tri-fluorescent viruses has allowed for the identification of the precise site of HIV-1 entry into cells to initiate the infectious lifecycle [7,9]. Such reporters have revealed an endosomal entry mechanism implemented by HIV-1 under live cell imaging conditions [7]. One such vector has been engineered to express a green fluorescent protein (GFP)-tagged nucleocapsid and a red fluorescent lipophilic dye, dialkylcarbocyanine analogue (DiD), which incorporates into the viral envelope [7]. Upon fusion between the virus and the host cell plasma membrane, both DiD and GFP-nucleocapsid are diluted within the excess amounts of lipids and cytosolic space located at the plasma membrane resulting in the abolishment of the red and green punctate fluorescence (Figure 1A) [7,10]. In contrast, when the viral particle is initially endocytosed before subsequent fusion with the endosomal membrane, the DiD label is retained on the endosomal membrane, and GFP-nucleocapsid is lost to cytosolic diffusion (Figure 1B). This technique has determined that HIV-1 virions enter into the cytoplasm and initiate infection following endocytosis. The identification of an endocytic entry mechanism for HIV-1 fundamentally shifted the established dogma of cell surface HIV-1 fusion, and viral movement through the endocytic pathway is now regarded as a key mechanism involved in HIV-1 entry [11]. Other variations of reporter viruses have also been used to visualize HIV-1 uncoating and disassembly following endocytosis. In particular, triple fluorescent viruses have been used to measure the rate of nucleocapsid, matrix and RNA trafficking following entry [9]. Moreover, dual/triple reporter viruses in cell culture models have enabled visualization of the site of entry into cells and provided time-lapse views of uncoating through single virus tracking within live cells [9].

Other innovative viral reporters uniquely exhibit properties of pH sensing, allowing the identification and elucidation of the pH-dependent steps involved in HIV-1 entry [8,12,13]. Indeed, this tool has enabled the identification of the early and late cellular endosomes as a location specifically implicated in HIV-1 entry. This technique involves the exploitation of the pH-sensitive GFP protein variant, pHluorin, fused to the N-terminal domain of intracellular adhesion molecule 1 (ICAM-1), which when expressed during virus production becomes incorporated within the viral membrane. Additionally, the expression of a pH-insensitive protein mCherry (red) is incorporated within the viral core through a Gag-mCherry fusion protein (Figure 1C). The simultaneous expression of pHluorin and mCherry corresponds to the endocytosis of the virion within a pH neutral compartment, whereas the exclusive expression of pHluorin reveals fusion directly at the cell surface [8]. Conversely, the exclusive expression of mCherry suggests fusion within the more acidic endosomal compartment due to the loss of pHluorin fluorescence. Importantly, pHluorin/mCherry demonstrated that viral entry from an acidic compartment specifically corresponded to the late endosomal stages and that endocytosed virions can often be shuttled back to the cell surface. While early seminal studies utilized transmission electron microscopy (TEM) to identify HIV-1 fusion at the cell surface [3], the advent of dually fluorescent HIV-1 virions and pH-sensing reporters have demonstrated that as much as 30% of virions can carry out fusion with endosomal membranes to initiate infections [8]. Overall, reporter viruses have become robust tools for deciphering the complexities of HIV-1 entry. 

## 3. Alternative Transmission of HIV-1: Cell–Cell Viral Entry

In contrast to canonical cell-free viral infection, HIV-1 entry also occurs through a direct cell–cell interaction, termed cell–cell transmission. In such instances a virological synapse is formed between infected and uninfected cells. In the simplest sense, a virological synapse is the point of contact between two cells, whereby HIV-1 is directly transferred from the infected to the uninfected cell. This is often a dendritic cell:T cell, macrophage:T cell, or T cell:T cell transmission [14,15,16]. Indeed, this transient event begins with binding of Env—the HIV-1 glycoprotein that serves to form the viral envelope—on the infected cell surface with CD4 on the uninfected cell surface, followed by recruitment of adhesion molecules, such as ICAM-1 to stabilize the synapse [16,17]. During these transient interactions, HIV-1 virions traffic from infected cells to infect the adjacent cell. Although the signalling events regulating this are not yet fully elucidated, the microtubule organizing centre (MTOC) can polarize to the synapse and likely plays an important role in synapse formation [18]. Furthermore, actin polymerization has also been reported at the virological synapse [16]. 

Synapses are not only observed between T cells, but may also form between antigen presenting cells (APCs) and T cells through the binding of the T cell receptors (TCRs) with cognate major histocompatibility complex (MHC) molecules on APCs, in which case the term immunological synapse is used [19,20]. Although immunological synapses generally utilize many of the same adhesion molecules as virological synapses, it is important to note that the initial receptors involved in synapse formation are distinct (TCR:MHC versus gp120:CD4, respectively) [20]. Interestingly, cell–cell transfer of viral particles between dendritic cells and T cells likely plays a major role in viral dissemination during HIV-1 infection [21]. Using live imaging microscopy, HIV-1 has been visualized to be recruited to the dendritic cell:T cell synapse [21,22]. It has been proposed that the active transfer of HIV-1 by dendritic cells to T cells amplifies the cytopathic effects of small amounts of virus [23]. Critically, not only does cell–cell transmission facilitate an increased rate and efficiency of viral transfer between cells as compared to cell-free transfer, but it also serves as a method of immune evasion through evading antibody neutralization of free virus and promoting the infection of target cells multiple times [24,25]. Although the ability of antibodies to access the synapse remains debated, it has been demonstrated that broadly neutralizing antibodies are less effective at inhibiting cell–cell transmission as compared to cell-free transmission [26]. Interestingly, the ability of broadly neutralizing antibodies to function is reported to be HIV-1 strain- and epitope-dependent, and contingent on the presence of these antibodies prior or post synapse formation [26,27]. Overall, because these interactions are short-lived, unique imaging techniques in cell culture models must be applied in order to visualize the synapse in action as part of the viral lifecycle.

### 3.1. Application of Co-Cultures to Study Cell–Cell Transmission

**Figure 2:**
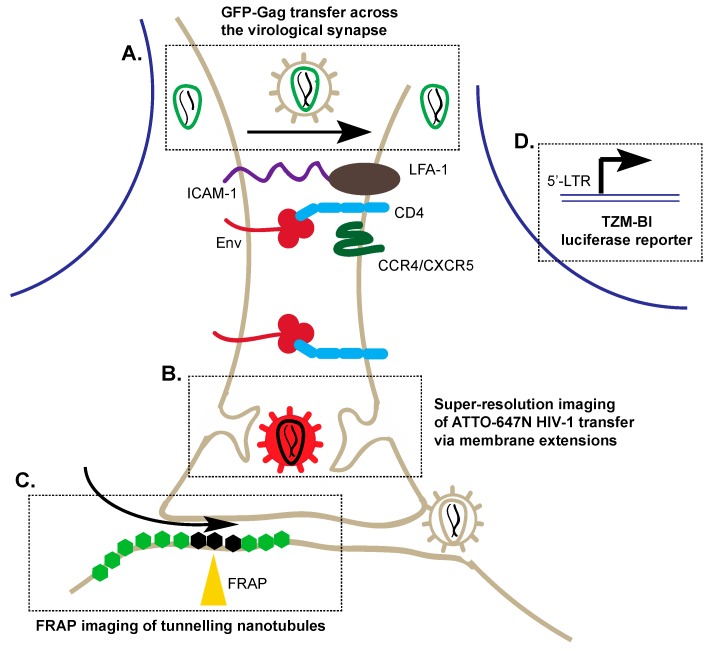
Techniques to visualize cell–cell transmission of HIV-1 through virological synapses and tunneling nanotubules. Upon engagement of Env with cluster of differentiation 4 (CD4) and C-C chemokine receptor type 4/ C-X-C chemokine receptor type 5 (CCR4/CXCR5), there is a recruitment of host cellular factors including ICAM-1 and leukocyte function-associated antigen 1 (LFA-1) to form the virological synapse. (**A**) 3D-confocal microscopy enables the visualization of GFP-Gag (**green**) particles across the virological synapse; (**B**) Super-resolution imaging of ATTO-647N (**red**) demonstrated that labeled HIV-1 can be taken up by a neighbouring cell though protruding membrane extensions within the virological synapse; (**C**) Fluorescence recovery after photobleaching (FRAP) studies enable imaging of the dynamics of membrane proteins in live cells to investigate the structure of tunneling nanotubules. Hexagons represent membrane proteins; bleached: **black**, fluorescent: **green**; (**D**) Co-culture of infected primary cells with TZM-Bl cells can be used to detect cell–cell transfer of HIV-1 by β-galactosidase activity, which is expressed under the regulation of the HIV-1 promoter; LTRs (long terminal repeats) are DNA sequences used by retroviruses to integrate into the host genome.

A combination of live cell imaging and co-culture techniques have been used to capture a glimpse of these cell–cell transmission events in real time [28]. This involves mixing two or more cell types in vitro—a process referred to as co-culturing—to dynamically examine inter-cellular interactions via microscopy. Typically, confocal and scanning electron imaging of co-cultures has allowed for visualization of virological synapse formation. For instance, Hubner et al. tracked the temporal movement of GFP-labelled HIV-1 Gag—the HIV-1 gene encoding the core structural proteins of the virus—from Jurkat cells to live CD4+ T cells, across the virological synapse [28]. Notably, Env-deficient virus could not induce cell–cell conjugation or Gag accumulation, demonstrating that cell adhesion occurs prior to Gag accumulation (Figure 2A) [28]. Scanning electron microscopy of the co-culture further illustrated ‘buttons’ at the cell surface containing oligomerized Gag protein which was eventually sequestered to form virus-laden internal compartments in target cells, suggesting transfer may be coupled with cellular endocytosis [28]. More recently the importance of Gag in synapse formation was also demonstrated by Gardiner et al. using live cell imaging of co-cultured infected COS-7 cells with uninfected Jurkat cells, illustrating that Env recruits large amounts of Gag to virological synapses, modulating synapse duration and stability [29]. However, further elucidation and clarification surrounding Gag and Env involvement in synapse formation is still required [29].

Overall, such co-culture and imaging techniques have contributed to elucidating that upon Env engagement of CD4 and CXCR4, there is an actin-dependent recruitment of adhesion molecules—mainly leukocyte function-associated antigen 1 (LFA-1), ICAM-1, and ICAM-3—and host/viral receptors—mainly CD4, CXCR4/5, Env, Gag— to the synapse [16,17,21]. Furthermore, imaging of co-culture synapse formation appears to be regulated via the kinase 70 kDa zeta-associated protein (ZAP-70) signalling pathway and recruitment of monosialotetrahexosylganglioside (GM1)-rich lipid-rafts to the cell membrane, as each are required for synapse formation in co-culture [30,31].

### 3.2. Super-Resolution Imaging of Cell–Cell Transmission

Although traditional confocal and electron microscopy provide detailed information on the distribution of proteins and cells that are required for cell-cell transmission, 3D cell imaging at a higher resolution truly visualizes viral trafficking events, including those involved in cell–cell entry. While sections or planes of TEM images can be recombined to generate detailed 3D landscapes of cells, this is laborious [32,33,34]. Rather, the emergence of stimulated emission depletion (STED) microscopy and ion abrasion scanning electron microscopy (IA-SEM) enables imaging of infected cells down to nanometer resolution.

The uniqueness of STED lies in its ability to image beyond the limits of diffraction. Historically, the resolution of microscopy has been limited to ~200 nm. Yet, STED overcomes these limits, implying single-virion resolution is now easily attainable [32,33,34]. Briefly, STED acquires images with sub-diffraction resolution by generating focal regions of molecular excitation which are smaller than the diffraction limit and quenching fluorescence of excited molecules via stimulated emission at the edge of the focal spot [32,33,34]. Conversely, IA-SEM exposes cells to gallium ion beams that sequentially remove material from the cell/specimen surface allowing each plane to be stuck with a scanning election beam [35,36]. This permits a 3D image to be reconstructed, mapping out the architecture of cellular structures in the nanometer range.

Combined use of STED and IA-SEM has demonstrated cell–cell transmission of HIV-1 between dendritic cells pulsed with ATTO-647N (a red spectrum fluorophore) labeled HIV-1 and CD4+ T cells labeled with anti-CD3 [14]. Strikingly, these techniques revealed that T cells are surrounded by membrane-like extensions originating from the originally infected dendritic cells and that extensions from CD4+ T cells themselves collect HIV-1 virions from surface-sequestered dendritic cell compartments during cell–cell transmission (Figure 2B) [14]. Notably, engagement of the CD4 receptor is required for viral transmission as synapse formation itself is insufficient to induce transfer of HIV-1 to uninfected cells [14]. IA-SEM has not only shed light on the formation of virological synapses but also on the intricacies involved in the transfer of virus through the synapse. For example, infected macrophages display a network of nanoscale tubes (~150–200 nm diameter, ~5 µm length) that relay internal viral reservoirs to the cell surface facilitating the ejection of HIV-1 virions onto uninfected cells [37]. 

### 3.3. Fluorescence Recovery after Photobleaching (FRAP)

Just as super-resolution imaging has provided previously undocumented detail on HIV-1 entry, equally important to advancing our understanding of cell–cell viral entry are techniques that capture the dynamic nature of these cell–cell interactions. One such technique incorporated to investigate cell–cell transmission of HIV-1 is fluorescence recovery after photobleaching (FRAP) which measures protein mobility in living cells. In brief, FRAP irreversibly quenches the signal of a fluorophore using a high intensity laser beam, after which non-bleached fluorescently labelled molecules can diffuse into and rescue the fluorescent signal of that region [38]. Rapid fluorescence recovery corresponds to increased protein diffusion. For example, FRAP bleaching of fluorescent anti-2G12 labelled Env has demonstrated that Gag assembly at the plasma membrane induces the aggregation of Env clusters into larger immobile domains [39]. It is proposed that such immobilization of Env prevents fusion of infected and uninfected cells at the virological synapse, and rather stabilizes virological synapse formation facilitating efficient cell–cell transmission of virus into target cells. Furthermore, FRAP of HIV-1 receptors has demonstrated that during viral entry CD4 and CCR5 are mobile on the plasma membrane, with CCR5 exhibiting a cholesterol-dependent increase in mobility when compared to CD4, suggesting that receptor mobility may affect receptor recruitment and binding of viral particles during viral entry [40]. 

In parallel, FRAP has characterized a form of HIV-1 cell–cell viral transmission termed ‘tunneling nanotubules’ (TNT) [41,42]. In this instance, a canonical viral synapse fails to form. Instead, thin connections up to several cell-diameters in length form between cells through which viral particles are transferred from infected to uninfected T cells [43]. Phosphatidylinositolbiphosphate (PIP2)—a phospholipid constituent of cell membranes—and Gag are localized in the distal nanotubule to make contact with the target cells, and tetraspanins—a family of transmembrane scaffolding proteins—are localized throughout the nanotubule structure [42]. FRAP of GFP-labelled Gag and GFP-phospholipase C (PLC)-labelled PIP2 in Jurkat cells demonstrates that Gag is immobilized on the plasma membrane and in TNT structures, as fluorescence recovery did not occur after bleaching, suggesting these are sites of virus-like particle (VLP) assembly (Figure 2C) [42]. Interestingly, PIP2, an important regulator involved in targeting of Gag to the plasma membrane, retained high motility and is not responsible for Gag immobilization [42]. Taken together, FRAP suggests that the TNT tip is another site of viral assembly and budding facilitating cell–cell transmission. Indeed, FRAP has demonstrated that these channels likely contain a junction, suggesting passage through TNTs is highly organized and regulated [42]. For example, bleaching of inner, outer and transmembrane anchored fluorescent proteins is able to be recovered within the TNT of T cells, suggesting plasma membrane proteins have access to the TNT [43]. However, these membrane proteins cannot diffuse into the adjacent cells, indicating a junction is present within TNTs, which may be involved in regulating virion or protein transfer between cells [43]. 

Indeed, HIV-1 viral particles have been separately located and imaged in nanotubules [41,44]. Using T cells fluorescently labelled with membrane dyes (dialkylcarbocyanine analogue (DiO), green; DiD, red), laser scanning confocal microscopy of live cells has revealed the presence of membranous tethers between cells, whereby HIV-1 is transferred to uninfected cells in a receptor-dependent manner [44]. An alternative form of cell–cell transmission involving filopodial bridges has also been reported in other retroviruses demonstrating that this is a conserved function [45].

### 3.4. Reporters to Examine Cell–Cell Transmission

Perhaps the most powerful tools utilized to understand cell–cell transmission have been reporter cell lines and viruses. A reporter is a gene that is placed under transcriptional control of a regulatory gene of interest. Of the reporter cell lines, the TZM-bl cell line is particularly useful in HIV research. Adapted from the JC.53 parental HeLa cell line—which expresses high levels of CD4 and CCR5—TZM-bl cells are susceptible to infection by a wide variety of HIV-1 isolates [46]. TZM-bl cells express β-galactosidase and luciferase under the control of the HIV-1 promoter, and hence enable selection and quantification of infectivity (Figure 2D). Abela et al. co-cultured infected peripheral blood mononuclear cells (PBMCs) with TZM-bl cells to monitor cell–cell transmission of HIV-1 and demonstrated that CD4 neutralizing antibodies are less potent inhibitors of cell–cell transmission when compared to cell-free transmission [47]. This suggests these antibodies act by inhibiting cell-free HIV-1 transmission and do not impede HIV-1 cell–cell transmission [47]. Hence, this in vitro cell reporter system has provided novel insight into how HIV maintains infectivity and evades antibody responses in vivo. 

The application of HIV-1 reporter viruses to study HIV-1 cell–cell transmission has also enhanced our knowledge of the HIV-1 lifecycle. Sloan et al. used a HIV-1 NL4.3-based GFP reporter virus which co-expresses Negative Factor (Nef) and GFP from a bicistronic RNA, whereby transmission was measured via integration of the virus into the genome resulting in GFP production [48]. It is known that cell-free HIV-1 can productively enter cells via dynamin facilitated clathrin-dependent endocytosis, however the mechanism of entry of HIV-1 into target cells across a virological synapse continues to be studied. Using this GFP-reporter virus, it has been demonstrated that viral entry into target cells during cell–cell transmission can occur via dynamin-dependent endocytosis leading to productive infection, and is a process which is sensitive to clathrin antagonists [48]. However, it was additionally found that HIV-1 can also choose alternate entry mechanisms, suggesting that the virus may be able to alter its entry path depending on the pressures it faces [48]. 

The range of these techniques—from co-culturing to reporter viruses—has allowed for investigations of cell–cell transmission, at each stage, from viral release by the infected cell to viral uptake by the uninfected cell across the virological synapse.

## 4. Preparing for Landing: Imaging HIV-1 Nuclear Entry and Integration

### 4.1. Imaging Reverse Transcription and Nuclear Entry

Following viral entry, a hallmark of the retroviral lifecycle is the ability to reverse transcribe the RNA genome into DNA, thereby enabling stable integration into the host’s genome [49,50]. As such, HIV-1’s ability to initiate reverse transcription and integrate into the host genome is critical for establishing chronic infection [51]. Recently, mechanisms of HIV-1 virion trafficking to the nucleus and integration have been unraveled using reporter viruses and RNA probes. 

**Figure 3:**
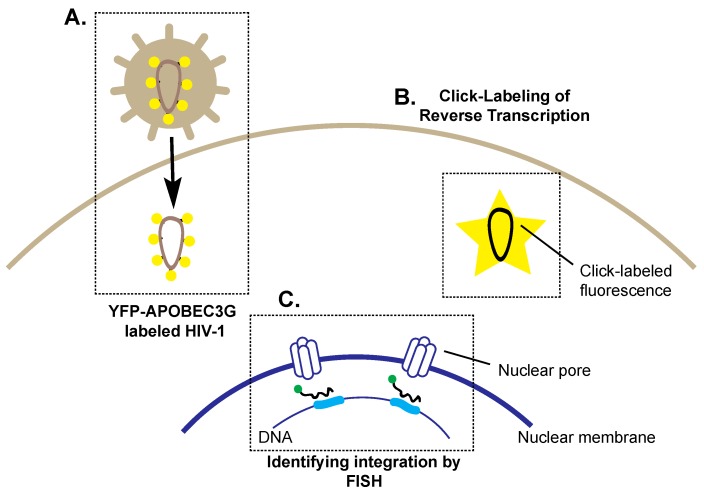
Selected imaging techniques and vectors used to identify key steps in HIV-1 trafficking to the nucleus. (**A**) yellow fluorescent protein-apolipoprotein B mRNA editing enzyme catalytic subunit 3G (YFP-APOBEC3G)-labeled HIV-1 virions (represented by **yellow** circles) demonstrate the trafficking of the nucleocapsid to the nuclear pore; (**B**) Click-labelling allows detection of reverse transcription in the infected cell, where the incorporation of 5’-ethynyl 2’-deoxyuridine into newly synthesized viral DNA results in detectable fluorescence, represented by the yellow star; (**C**) The use of fluorescence in situ hybridization (FISH) probes (**black** with **green** fluorophore) identified that preferential integration sites (**blue**) are located near nuclear pores.

The process of reverse transcription occurs in close association with the nucleocapsid and requires host and viral proteins including reverse transcriptase, integrase and the accessory protein viral protein R (Vpr) [52,53,54,55,56]. Combined, this complex can be termed the reverse transcriptase complex (RTC) or the pre-integration complex (PIC), depending on whether or not reverse transcription (RT) has occurred [57]. For viral genome integration, the PIC must localize to the nucleus in order to prime the cell [55]. Innovative cell culture techniques have provided novel information on HIV-1’s ability to integrate host chromosomal DNA [58]. Burdick et al. tracked PIC using viral particles produced from cells expressing the HIV-1 restriction factor apolipoprotein B mRNA editing enzyme catalytic subunit 3G APOBEC3G in a yellow fluorescent protein (YFP)-tagged form [59]. APOBEC3G is a cysteine deaminase which incorporates into the virion and deaminates cytidine residues to uridine resulting in the high inefficiency of reverse transcriptase [60]. YFP-APOBEC3G-labeled virions demonstrated that reverse transcription was not required for nuclear import, but rather that capsid protein stability was a predictor of the correct nuclear pore localization of the PIC (Figure 3A) [59]. Such studies implementing a Vpr-GFP fusion, which is also actively incorporated into the virion, have also demonstrated the perinuclear localization of the PIC. As such, YFP-APOBEC3G and Vpr-GFP virions unveiled early nucleocapsid trafficking events during the HIV-1 lifecycle. 

A challenge of studying early HIV-1 lifecycle stages remains the direct visualization of reverse transcription within the cell. PCR techniques have detected different stages of RT [61,62,63], however they cannot detect the temporal and spatial scale of RT activity. Recently it has been demonstrated that RT activity can be directly measured in cells using “Click labelling” which overcomes the functional limitations of labeling RNA and DNA. Click labelling was originally described by Salic and Mitchison, and labels newly synthesized DNA through the incorporation of 5’ethynyl 2’-deoxyuridine into viral DNA, which allows for fluorescence detection by microscopy (Figure 3B) [64]. Remarkably, this enables the detection of reverse transcription within the cell, as it labels newly synthesized DNA in the cytoplasm. Click chemistry, in combination with immunofluorescence, revealed how the PIC/RTC remains associated with the capsid during nuclear trafficking and reverse transcription, a phenomenon long believed to occur independently from the nucleocapsid [65]. Indeed, this study has solidified the importance of the capsid in both pre- and post-nuclear entry lifecycle events.

### 4.2. Imaging Integration and Latency

The process of integration initiates the start of a productive infection to generate new progeny virions [66]. The ability of viral DNA to integrate into the genome establishes latency during the HIV-1 lifecycle, whereby cells contain integrated viral DNA, termed provirus, but are not actively transcribing viral proteins [67]. The establishment of latent reservoirs is one of the greatest barriers to eliminating the virus [68]. To effectively develop therapeutics targeting these latent reservoirs, an understanding of how and where HIV-1 prefers to integrate within host genomes is necessary.

The re-emergence of fluorescence in situ hybridization (FISH) has shed light on the precise nuclear localization of viral DNA integration [58,69]. FISH utilizes fluorescent DNA oligomers to locate DNA, which has been used to successfully locate integrated viral genomes (Figure 3C) [69]. Recent work has examined the localization of proviral DNA within the nucleus [58]. Previous studies had largely focused on HIV-1’s ability to integrate within certain gene types [70,71]. In general, it was believed that HIV-1 integration prefers transcriptionally active genes [71], however no prior studies had identified a consensus sequence for integration. An examination of integration site data from infected individuals revealed a set of integration site hot spots which have been termed the recurrent integration genes (RIGs). Multiple FISH probes in combination with 3D confocal microscopy revealed the position of the RIGs in relation to the nuclear envelope and nuclear pores [58]. Interestingly, many of the genes are in close proximity to the nuclear envelope, suggesting that it may not be the sequence which determines integration, but rather the DNA’s location within the nucleus [58]. A similar analysis with HIV integration sites revealed a preferential integration near nuclear pores [72]. This seminal study highlights the importance of combining older techniques such as FISH with newly emerging imaging techniques to uncover previously unobserved intricacies of HIV-1 integration. 

## 5. Trespassing into Enemy Territory: Imaging Host Cell Hijacking by HIV-1 Nef

Subsequent to HIV-1 entry into cells, the reverse transcribed viral nucleic acid is targeted to the nucleus whereby following integration, the transcription and translation of new viral proteins facilitate virus assembly and propagation of the HIV-1 lifecycle. A mechanism that ensures the HIV-1 lifecycle is safeguarded is the production of the HIV-1 accessory proteins, viral infectivity factor (Vif), Vpr, viral protein unique (Vpu), and Nef which work to hijack the host cell [73]. Primarily, Vpu, Vif, and Vpr selectively degrade host cell proteins which interfere with viral replication [73]. Perhaps the most elusive of all HIV-1 usurping functions is the ability of Nef and Vpu to downregulate immune receptors from the cell surface to prevent immune recognition of infected cells [73]. Again, the use of numerous cell culture-based imaging techniques have unveiled the intricacies of viral hijacking of host cells by accessory proteins throughout the viral lifecycle.

Specifically, we and others have demonstrated that Nef manipulates the host cell membrane trafficking machinery to downregulate multiple cell surface receptors, including CD4 and MHC-I [74,75,76]. Indeed, the downregulation of MHC-I from the cell surface by Nef is a central host cell-hijacking event of HIV-1 infection (reviewed by Pawlak and Dikeakos [74]). Correspondingly, Nef trafficking and function depend extensively on overtaking a plethora of host cellular proteins, resulting in a multitude of viral–host interactions within the HIV-1 lifecycle, and will serve as the model for the techniques discussed below.

### 5.1. Forster Resonance Energy Transfer

**Figure 4:**
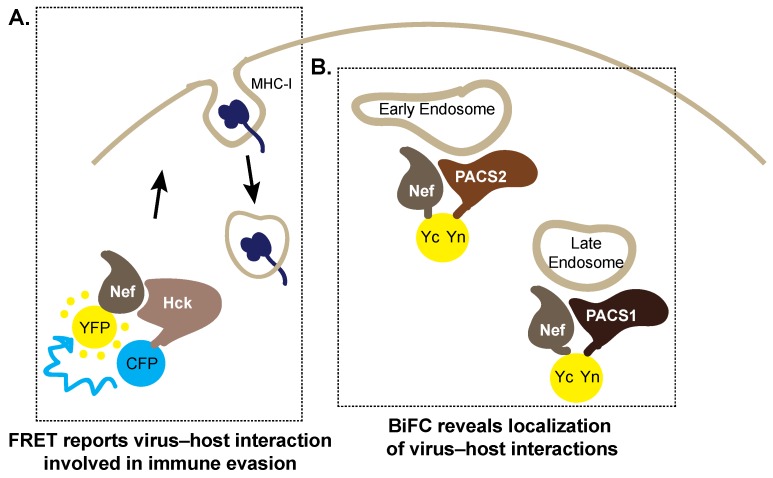
Fluorescence-based methods to monitor virus–host interactions required for the HIV-1 accessory protein negative factor (Nef) to hijack the host cell. (**A**) Key interactions between Nef and the Src-family kinase Hck are detectable in cells by Forster resonance energy transfer (FRET). Co-expression of Hck–CFP (**cyan** fluorescent protein) and Nef–YFP (**yellow** fluorescent protein) generates a FRET signal demonstrating their interaction. This interaction activates the signaling pathways required to internalize major histocompatability complex-I (MHC-I) from the cell surface to prevent cytotoxic killing of HIV-1 infected cells; (**B**) Bimolecular fluorescence complementation (BiFC) reports the interaction between the PACS1 and PACS2 proteins and Nef, localizing these interactions to early and late endosomal compartments, respectively. Yn: N-terminal of YFP, Yc: C-terminal of YFP, which reconstitute upon protein–protein interactions.

In order to catch a glimpse of such events in cell culture, the use of Forster Resonance Energy Transfer (FRET) has emerged as a valuable microscopy technique. Briefly, a FRET signal is a distance-dependent phenomenon whereby energy is transferred between molecules, suggestive of a protein-protein interaction [77]. Specifically, two proteins of interest are tagged with a cyan fluorescent protein (CFP) and YFP, respectively. If these proteins are in close proximity (within 10–100 Angstroms), upon laser excitement of the CFP, energy will relay from the excited donor fluorophore (CFP) to the acceptor fluorophore (YFP) producing a YFP signal [77,78]. One elegant application of FRET has been in elucidating the membrane trafficking cascades that contribute to immune evasion by HIV-1. Hung et al. [79] reported that Nef assembles a Src Family kinase-ZAP-70/Syk-phosphatidylinositol-4,5-bisphosphate 3-kinase (PI3K) complex to downregulate MHC-I, and utilized FRET to demonstrate the interaction of Nef with the Src family kinase Hck (Figure 4A). Although FRET microscopy often involves energy transfer from CFP to YFP, other combinations of fluorophores or molecules can be used as long as their emission spectra overlap. This is highlighted by Gerlach et al. who used FRET to complement their studies characterizing HIV-1 Nef binding to membranes [80]. In this case, the intrinsic tryptophans of Nef were excited and the energy relayed to a diphenyl-based fluorescent dye immersed in the lipid bilayer of isolated liposomes to generate a FRET signal, serving as a model to measure Nef binding to membranes [80]. Although this FRET technique was completed in vitro, it would be interesting to apply it in situ to investigate Nef membrane interactions at the cellular level.

### 5.2. Bimolecular Fluorescence Complementation

The host–cell membrane trafficking machinery commandeered by Nef to shuttle itself throughout the various compartments of the cell has also been extensively investigated. Interestingly, this unraveled a new class of membrane trafficking regulator proteins, referred to as the phosphofurin acid cluster sorting proteins (PACS), being involved in Nef transport [81,82]. In fact, is has been shown that PACS2 binds the Nef EEEE_65_ motif to target the Nef:PACS2 complex to the paranuclear region whereby the Nef PxxP_75_ region interacts with Src family kinases (SFKs) at the trans Golgi network (TGN) to bind and activate the SFKs [81]. This complemented the previously reported Nef:PACS1 interaction involved in shuttling internalized MHC-I to the TGN [82]. Furthermore, these reports sparked interest to further elucidate the molecular interactions required for assembly of the multikinase cascade required for MHC-I downregulation, a question which was tackled using bimolecular fluorescence complementation (BiFC). BiFC generates a fluorescent signal after two non-fluorescent fragments of a split fluorophore fused on two distinct proteins of interest come within proximity to reconstitute a functional fluorophore, and represents a valuable method for studying virus–host protein–protein interactions [83]. Uniquely, the distance required for fluorophore reconstitution is ~20 Angstroms in BiFC. 

To further implicate the Nef–PACS interactions involved in MHC-I downregulation, it was demonstrated that the furin binding region (FBR) of PACS1 and PACS2 interacted with a bipartite site on Nef [84]. This bipartite site consisted of the N-terminal EEEE_65_ acidic cluster and the tryptophan 113 residue in the core domain of Nef, respectively [84]. Indeed, Nef and PACS1 or PACS2 fused to split yellow fluorescent protein fragments (Nef–Yc and PACS1–Yn or PACS2–Yn, where Yn: N-terminal of YFP, Yc: C-terminal of YFP) generated a BiFC signal within cells (Figure 4B) [84]. Strikingly, aberration of this Nef–PACS interaction also reduced MHC-I downregulation [84].

We have also applied BiFC to elucidate the intracellular vesicular trafficking pathways hijacked by the HIV-1 accessory protein Nef—a novel application which we term viral BiFC [85]. Although Nef interactions throughout the viral lifecycle have been previously investigated using in vitro assays such as co-immunoprecipitation studies and mass spectrometry, these methods did not evaluate Nef interactions in the context of a viral infection and did not provide information regarding the subcellular localization of such Nef interactions. Hence, we generated a lentiviral BiFC expression vector using the foot-and-mouth disease virus 2A cleavage site allowing for simultaneous expression of Nef and any host cellular protein of interest from a single transcript. These BiFC studies confirmed the previously reported interaction between the host membrane trafficking protein, PACS1, and Nef [84,85]. This technique further demonstrated that the Nef:PACS1 complex localized to lysosomal-associated membrane protein 1 (LAMP-1) positive endolysosomal vesicles, solidifying the role of PACS proteins involved in shuttling Nef throughout the cell to exert its hijacking function [85]. Furthermore, a Nef:MHC-I interaction was visualized and found to be localized to adaptor protein-1 (AP-1) positive compartments, providing additional evidence for the involvement of AP-1 in MHC-I internalization from the cell surface [85]. Hence, using BiFC, spatiotemporal information can be acquired in addition to determining viral-host interactions, something which strengthens our understanding of the dynamic nature of the HIV-1 lifecycle.

### 5.3. Ground State Depletion Super-Resolution Microscopy

In the same manner that the methods described above are implemented using conventional wide-field microscopes, super-resolution imaging can be introduced to generate detailed images of subcellular interactions and complexes. In particular, ground state depletion (GSD) microscopy captures images with a lateral resolution of up to 20 nanometers; a resolution 10× greater than conventional microscopes [86]. This technique is rooted in the ability to stochastically switch fluorophores between dark and fluorescent states at low densities [86,87]. This allows for a precise mapping of the position of active fluorophores during each acquisition phase, via mapping a Gaussian function to the point-spread function of each fluorophore, to generate a high-resolution image [86]. 

We have applied GSD microscopy to image the viral protein interactions, dynamics, and multi-protein complexes modulated by Nef during HIV-1 cell hijacking. Uniquely, by implementing the recently developed Molecular Interactions in Super-Resolution (MIiSR) software, intermolecular interactions and higher-order molecular complexes were quantified on super-resolution images [88]. Notably, the expression of Nef and the membrane trafficking regulator PACS1, revealed a significant amount of Nef in complex with PACS1 [88]. Importantly, this revealed temporal changes of complexes within cells, as cells imaged over varying time points revealed that the proportion of HIV-1 Nef interacting with PACS1 is reduced over time, consistent with its previously reported change in function over time, of switching from altering endocytosis to altering Golgi export [89]. Hence, super-resolution microscopy can capture changes in the individual components and proportions of molecular complexes formed during viral hijacking of host cells over the course of a viral lifecycle. Furthermore, the higher resolution of GSD microscopy validates protein colocalization observed at lower levels of resolution obtained with conventional wide-field microscopy. As such, the Nef-host interactions reported using BiFC have been validated at the super-resolution level using GSD microscopy [90]. 

Indeed, combining the methods above has allowed for elucidating the specific hijacking events by Nef to facilitate receptor downregulation over the course of the HIV-1 infectious cycle. Through microscopic imaging of viral-host interactions in culture, the trafficking and signaling events of HIV-1 Nef involved in receptor downregulation have been characterized [74]. 

## 6. The Great Escape: Imaging HIV-1 Assembly and Budding

### 6.1. Visualizing RNA Trafficking to HIV-1 Assembly Sites

Efficient trafficking of specific host and HIV-1 viral proteins to the cell surface is a requirement for dissemination of infectious virions [91]. HIV-1 Gag is the primary structural component of HIV-1 and is activated by HIV-1 protease following new virus budding [91]. Gag assembly is dependent on its homodimerization within the cell followed by multimerization at the surface [92]. Interestingly, Gag buds independently of the viral genomic RNA to form virus-like particles [93]. However, to kick-start subsequent rounds of infection, the proper localization of the viral genomic RNA to cell surface by assembled Gag is required. In the section below, we outline cutting-edge imaging techniques available to investigate viral assembly and egress in cell culture, several of which led to seminal discoveries surrounding viral assembly and budding.

**Figure 5:**
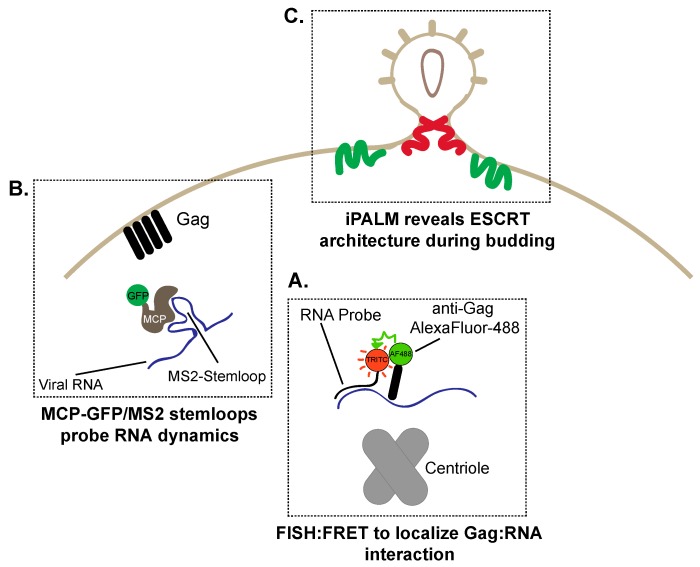
Tools to study HIV-1 assembly and budding. (**A**) Gag and viral RNA has been localized to the centriolar region by visualizing FRET between a FISH probe (tetramethylrhodamine (TRITC):**Red**) targeted towards viral RNA and AlexaFluor-488 immunostained Gag (**Green**); (**B**) Gag multimers assemble on the plasma membrane prior to viral RNA localization. RNA dynamics can be visualized by exploiting the high affinity interaction between the major capsid protein (GFP (**Green**)–major capsid protein (MCP; **Brown**)) and an MS2 bacteriophage stem-loop engineered onto the HIV-1 genome (**C**) Super-resolution interferometric photoactivation and localization microscopy (iPALM) imaging demonstrates how endosomal sorting complexes required for transport (ESCRT) proteins assemble around HIV-1 budding sites. Charged multivesicular body protein 2a/4b (CHMP2A/4B) (**Red** and **Green**) assemble within the neck of the budding virion to enable pinching of the plasma membrane to form single virions.

Early experiments exploring RNA–Gag interactions relied on FRET using a combination of immunofluorescence and in situ hybridization of RNA [94]. A combination of tetramethylrhodamine (TRITC)-immunostained Gag and AlexaFluor-488-conjugated antisense-RNA directed at HIV-1 viral RNA demonstrated the interaction between viral RNA and Gag by FRET (Figure 5A) [94]. Critically, this interaction was centered around the centriolar region [94]. Furthermore, the precise localization of viral RNA and the RNA–Gag interaction was dependent on the psi sequence, a specific region in the viral RNA necessary for the in vitro interaction [94]. Dynamics of the Gag–viral RNA interaction were established under live cell imaging [95]. To track viral RNA, the incorporation of the *Escherichia coli* anti-termination beta-glucoside utilization gene product (BglG) protein in an YFP-tagged form into the *Pol* region of the HIV-1 genome detected single RNA viral genomes. This technique is based on the high affinity interaction of BglG protein and RNA which contains a specific recognition sequence [96]. Analysis of YFP-BglG-tagged genomes revealed that disruption of the cytoskeleton did not alter the random walk nature of the RNA [95]. From this, it was established that the random nature of RNA trafficking within the cell, independent of host factors, may represent a mechanism where HIV-1 ensures delivery of viral RNA for assembly with minimal evolutionary push back from the host to ensure the successful completion of the viral lifecycle. 

One prominent technique, total internal reflection microscopy (TIRF), has further enhanced the study of viral assembly. TIRF allows the direct visualization of molecules which are at or in close proximity to the cell surface [97]. Whereas conventional confocal microscopy fails to reliably acquire the cell surface architecture due to the subjective nature attributed to the determination of the focal plane [97], TIRF allows the direct imaging of proteins which are within 100–250 nm of the cell surface [97]. Importantly, TIRF can be coupled with live cell imaging to study dynamic processes at the cell surface. TIRF microscopy distinctly illuminates the specimen at an angle and refracts light due to differences between numerical aperture (NA) of the coverslip (NA ~ 1.4) and the specimen (NA ~ 1.2). This feature lends itself perfectly to study HIV-1 assembly and budding. Indeed, the combined use of TIRF and super-resolution imaging has characterized HIV-1 assembly and release at the cell surface, and has been instrumental in uncovering the mechanism of viral budding [98,99]. One of the first comprehensive live cell analyses of viral assembly sites was conducted by Jouvenet et al. [99,100] who demonstrated Gag assembly at the cell surface using GFP-tagged Gag. Live cell TIRF imaging revealed that Gag assembled in small clusters representing unique virions. This broke the previously established dogma of random Gag budding occurring in non-discrete regions of the plasma membrane [93].

Similar techniques were also implemented to identify how viral RNA was trafficked to the budding virion [100]. Since many live cell imaging techniques often rely on fluorescently tagged proteins, visualizing RNA within cells is often hindered by the limitation of imaging fixed cells [101]. To overcome this limitation, the study of RNA dynamics within live cells is possible by exploiting the high affinity interaction between the MS2 bacteriophage major capsid protein (MCP), and the MS2 stem-loop found in the bacteriophage [102]. In brief, the MCP–MS2 system requires the expression of GFP-tagged MCP in combination with the mRNA of interest containing an MS2 stem-loop motif within the 5’ untranslated region. Upon expression of GFP-tagged MCP and MS2-tagged viral RNA, the high affinity interaction efficiently labels the viral RNA, thus enabling the live cell imaging (Figure 5B). The MCP–MS2 system defined how viral RNA is successfully incorporated into virions at the plasma membrane [100]. In addition, TIRF microscopy revealed the high degree of viral genomic RNA mobility in the absence of Gag and conversely revealed immobilized viral RNA in the absence of Gag, suggesting that Gag is required for RNA to be stabilized at the plasma membrane [100]. Interestingly, MS2-tagged RNA assembled on a diffuse Gag-mCherry assembly location and recruited additional Gag multimers to the assembly site. This hints at a novel mechanism requiring viral RNA to facilitate Gag assembly. Overall, the demonstration that the HIV-1 viral genome is both stationary and motile provides key insight into the assembly of infectious virions.

### 6.2. Super-Resolution Imaging of HIV-1 Budding

As obligate intracellular parasites, viruses require host cellular proteins to carry out specific functions to complete the virus lifecycle. HIV-1 relies heavily on a subset of proteins known as the endosomal sorting complex required for transport (ESCRT) machinery [103]. The ESCRT machinery is divided into three groups, ESCRT I and II are involved in binding of cargo molecules, and act as building blocks for ESCRT III, which consists of factors mediating membrane bending, remodeling and vesicle pinching [104]. The ESCRT machinery is largely responsible for the inward invagination of vesicles into multivesicular bodies, and specific knockdowns of ESCRT proteins inhibit HIV budding from the cell surface [105]. Early microscopy studies demonstrated that the HIV structural protein Gag recognizes ESCRT at the cell surface [106]. Prior to this, it was believed that HIV budding was spontaneous and required few host factors.

Traditionally, a major obstacle in defining molecular mechanisms of viral exit and assembly has been the limit of resolution of conventional microscopy, as wide-field and confocal microscopy resolve molecules ~180 nm apart in perfect conditions [107]. Recent studies using super-resolution imaging have shed light on the assembly of cell surface ESCRT/Gag complexes [108,109]. In particular, the combination of electron and super-resolution microscopy to generate a technique known as 3D correlative interferometric photoactivation and localization microscopy (iPALM) has enabled the precise mapping of the cellular architecture for the ESCRT proteins mediating budding (Figure 5C). iPALM takes advantage of the on and off fluorophore switching to maximize resolution [110]. However, unlike GSD, iPALM achieves <50 nm resolution in the *x*, *y* and *z* planes. Briefly, iPALM resolves the *z* plane by evaluating the time required for emitted light originating from the sample to reach a detector [110]. Using this technique, valuable information has been gained on the architecture of the viral budding process. It was demonstrated that ESCRT-I tumor susceptibility gene 101 (tsg101) and ESCRT-III proteins vacuolar protein sorting-associated protein 4 (Vps4), charged multivesicular body protein 2a/4b (CHMP2A/4B) localize to budding Gag virus-like particles [109]. Additional experiments utilizing a combination of correlative iPALM, two-color iPALM and TIRF were used to study HIV-1 viral assembly. These high-resolution techniques revealed that fluorophore-tagged tsg101, Vps4 and CHMP2A/4B localized to the neck of the virion bud, implying that the assembly of ESCRT complexes participates in virion budding from the plasma membrane [109]. Using similar techniques, endogenous CHMP4B and CHMP2A were also localized to the neck of the budding virion [111]. Moreover, these techniques positioned ALG-2 interacting protein X (ALIX), a member the ESCRT III, directly into budding HIV-1 virions where it would act as a host protein implicated in the outward mechanical movement of virions [111].

Taken together, it is clear that the combination of super-resolution imaging and live cell TIRF microscopy has revealed fascinating details on the cellular machinery governing HIV-1 assembly and budding at the plasma membrane. The highly resolved images of the ESCRT machinery at the cell surface by iPALM exquisitely highlights the capabilities of emerging techniques to HIV-1 research as a whole. 

## 7. Applications of Cell-Culture Based Microscopy Assays for Drug Discovery

As the global push to eradicate AIDS continues, microscopic techniques in partnership with cell culture have been implemented to screen for novel AIDS therapeutics. For instance, wide-field imaging of viral or host proteins in the presence of potential HIV-1 inhibitors provides subcellular spatiotemporal information regarding the effectiveness of such therapeutics. Gu et al. were able to chemically inhibit the interaction between HIV-1 integrase and the lens epithelium-derived growth factor (LEDGF/P75) complex, a transcriptional co-activator of integrase used for incorporating the correct viral genome into the host DNA [112]. Compound D719 blocked this interaction and the nuclear entry of integrase, as visualized by immunofluorescent imaging of the inability of GFP-labeled integrase to translocate into the nucleus [112]. 

Moreover, high-throughput screening and cell imaging techniques are in continuous development to rapidly evaluate the efficacy of HIV-1 inhibitors in cell culture. Notably, an automated image-based assay to evaluate antibody neutralization of HIV and inhibition of cell–cell fusion has been implemented allowing screening for neutralizing antibodies which may act as inhibitors of viral entry [113]. Particularly, in line with attempts to develop an effective HIV-1 vaccine, part of the evaluation process is the assessment of the neutralizing capacity of antibody responses. Unfortunately, no single assay detects such protective antibodies. U87.CD4 or GHOST(3) cells expressing HIV receptors have been exposed to HIV-1 and imaged for plaque formation and plaque area using automatic image acquisition in an automated plaque reduction assay [113]. In this case, antibody neutralization of free virus is indicated via a reduction in plaque number and a reduction in plaque area corresponds to an inhibition of cell–cell fusion (syncytia) which is characteristic of infected cells [113]. Computational analysis quantifies the number of plaques and plaque area, giving high-throughput analysis of therapeutic efficacy.

Similar high-throughput screening assays for inhibitors of cell hijacking have also been developed. Rapid imaging of BiFC interactions can be implemented to screen for small-molecule inhibitors of HIV-1 Nef dimerization, which plays a role in its function of receptor downregulation [114]. In this case, Nef fused to fragments of YFP will reconstitute upon Nef dimerization. Automation is achieved through expressing Nef-YFP and a red fluorescent protein (RFP) reporter from the same vector in cell culture [114]. Screening revealed several small molecules which could inhibit Nef dimerization and subsequently play a role in Nef function. Inhibiting such interactions may add to efforts to reduce viral pathogenesis during the HIV-1 infectious cycle.

Interestingly, a genome wide-RNAi screen has been used to identify genes which play a role in blocking HIV infection. In this case, after a microarray-based siRNA knockdown, cells are automatically imaged using confocal microscopy [115]. Multiple imaging parameters can be captured simultaneously including cell number, morphology and the distance between neighbouring cells. Multidimensional visual profiling was then used to determine individual genes that supress HIV-infection [115]. This automated approach identified 56 host genes that generated CD4-like phenotypes upon infection, 45 of which were not previously known to be associated with HIV infection [115]. 

Moving forward, implementing such rapid screening and high-throughput imaging techniques will be essential to elucidate the complexity of host-viral interactions, and evaluate the potential of novel therapeutics in cell culture. 

## 8. Future Directions

Despite the plethora of imaging assays that currently exist for capturing glimpses of viral lifecycles, there continue to be areas in which opportunity for advancement exists. Arguably, super-resolution imaging has enhanced our visualization, and correspondingly understanding of virus–host interactions. However, the majority of these images have been obtained from fixed cells. Hence, there is an under-representation of imaging reports describing temporal and transient viral-host interactions within the literature. Optimistically, many of these forms of super-resolution techniques such as STED and PALM can image live cells, although rates of acquisition remain limited [116]. Regardless, with the advent of highly sensitive cameras, and improved acquisition parameters, it is likely that visualizing the full infectious cycle of any viruses in live cell super-resolution is well within future reach [117]. 

As the limits of resolution continue to be broken, now is perhaps the time to take a step back and examine how cells move and behave on a macroscopic level in order to confirm in vitro findings. Although cell culture models represent an easy method to visualize virus–host interactions, the advancement of ex vivo and multi-photon intravital imaging mark the next level of microscopy [118]. Remarkable studies have demonstrated the migration of HIV-1 infected T cells and the formation of virological synapses. These studies directly support multiple cell culture models of HIV-1 cell to cell transfer [118]. In addition, ex vivo and 3D cell culture models have provided invaluable information on T cell migration and viral dissemination [119]. Continuing to build on these seminal studies with advances in high resolution imaging will only strengthen our understanding of the complexities of the HIV-1 lifecycle. 

## 9. Conclusions

Endeavors to generate assays to visualize HIV-1 have served as the foundation for the development of the cell culture imaging techniques discussed in the current review. The implementation of cellular and viral reporters, as well as super-resolution imaging in conjunction with co-cultures, have not only demonstrated cell-free entry of HIV-1 entry into cells, but are also responsible for discovery of the previously unobservable cell-cell transmission of HIV-1. In addition, the development of FISH and APOBEC-3G YFP virus to image nuclear trafficking of HIV, and Click fluorescent labeling of nucleic acids to image reverse transcription have provided a direct visualization of the critical events involved in establishing a chronic HIV-1 infection. Largely, the generation of fluorescence upon protein–protein interactions via FRET and BiFC, as well as the implementation of GSD super-resolution microscopy have elucidated several HIV-1 cellular hijacking events. Additionally, TIRF, PALM and fluorescent RNA tracking have determined the sequence of events involved in HIV-1 assembly and egress. Importantly, although the techniques discussed within this review have been explained for HIV-1, they can potentially be applied to any virus of interest. However, such imaging may be of particular interest to apply to emerging viruses, for which lifecycle information needs to be further elucidated.

Several of the approaches reviewed above (BiFC, FRET and GSD) are based on the use of protein-fluorophore fusions to determine protein–protein interactions. Although the use of fusion proteins is well accepted, they may alter protein function and proper controls should be implemented to ensure function has not been disrupted [120]. When protein function may be compromised, tools such as the bis-arsenical fluorescein derivative, FlAsH, can be used to track the kinetics HIV-1 integrase within the cell [121]. In this method, proteins are engineered to contain a tetracysteine motif which can be bound by fluorescent FlAsH-EDT2 (ethanedithiol) to track protein-protein interactions [121]. Alternatively, SNAP-tag labels proteins with a non-fluorescent tag (SNAP-tag) can be used which will covalently couple to fluorescent molecules, such as O^6^-benzylguanine-tetramethylrhodamine-Star (BG-TMR-Star), facilitating visualization [122]. SNAP-tagged HIV-1 Gag has been used to visualize HIV-1 interactions in the cell as an alternative to conventional fluorophore fusion proteins [122]. Moreover, multiple findings reported in microscopy studies largely involve immortalized cell lines and this is also the case for imaging HIV-1. As such, prudent approaches should be undertaken to ensure the biology is translated in more physiological cell types, such as primary cells isolated directly from the organism [123].

Overall, the implementation of cell culture-based imaging techniques provides great promise for advancing and evaluating therapeutics for HIV-1 treatment. In particular, high-throughput automated microscopy techniques represent a rapid method of screening for inhibitors of HIV-1 lifecycle stages, and assessing vaccine effectiveness. However, perhaps the greatest impact of these imaging techniques are the pure visualization of viral lifecycle events that would have never been uncovered without their use. Above all else, it is these imaging techniques that have allowed us to explore deep within the depths of the cell, exposing a complex molecular universe.
